# Up-to-the-Minute Privacy Policies *via* Gossips in Participatory Epidemiological Studies

**DOI:** 10.3389/fdata.2021.624424

**Published:** 2021-05-13

**Authors:** Aakash Sharma, Thomas Bye Nilsen, Katja Pauline Czerwinska, Daria Onitiu, Lars Brenna, Dag Johansen, Håvard D. Johansen

**Affiliations:** ^1^Department of Computer Science, UiT The Arctic University of Norway, Tromsø, Norway; ^2^Faculty of Design, Computer Science, Media, RheinMain University of Applied Sciences, Wiesbaden, Germany; ^3^Northumbria Law School, Northumbria University, Newcastle upon Tyne, United Kingdom

**Keywords:** compliance, privacy, big data processing, research data, privacy policies, gossip, data sharing, open data

## Abstract

Researchers and researched populations are actively involved in participatory epidemiology. Such studies collect many details about an individual. Recent developments in statistical inferences can lead to sensitive information leaks from seemingly insensitive data about individuals. Typical safeguarding mechanisms are vetted by ethics committees; however, the attack models are constantly evolving. Newly discovered threats, change in applicable laws or an individual's perception can raise concerns that affect the study. Addressing these concerns is imperative to maintain trust with the researched population. We are implementing Lohpi: an infrastructure for building accountability in data processing for participatory epidemiology. We address the challenge of data-ownership by allowing institutions to host data on their managed servers while being part of Lohpi. We update data access policies using *gossips*. We present Lohpi as a novel architecture for research data processing and evaluate the dissemination, overhead, and fault-tolerance.

## 1. Introduction

Data-driven research on human subjects often requires *informed consent* from participating individuals before data can be collected and processed (Schneider, 2019). A responsible data scientist must therefore build on the notion of accountability where sensitive data of subjects are meticulously handled (Litton, 2017; Shneiderman, 2020) in accordance with governing laws and regulations. Although institutional ethics committees are tasked by the World Health Organization (2009) to protect subjects from anticipated harm, they have few means to do so beyond the initial project approval phase. Common mechanisms recommended for protecting data throughout a project's lifetime, such as anonymization and aggregation, have known limitations that have led to privacy violations (Kroll et al., 2019; Cummings and Durfee, 2020; McGraw and Petersen, 2020). More advanced privacy-enhancing mechanisms, such as based on the notion of differential privacy (Dwork et al., 2006) might protect data for the project duration, but are difficult to make use of in many scenarios (Kroll et al., 2019) and are too restrictive. Yang et al. (2012) summarized issues with differential privacy in data management involving large scale personal information. It is difficult to design differential privacy protocols for handling updates. The existing differential privacy studies assume a simple data model and centralized database. They are not feasible for already collected research data that lies in federated databases at multiple trustworthy research environments. Recent works, such as Garfinkel et al. (2018) and Suriyakumar et al. (2020) have raised the concerns, such as limited access to micro-data, misunderstandings about randomness and noise, and accuracy in using differentially private machine learning on healthcare data.

Another aspect of data-driven research is growing collaboration on the use of datasets. Goodman and Meslin (2014) and Salerno et al. (2017) highlight the challenges and goals for data sharing in epidemiology in particular. This includes the problems we find in different areas of big-data computing on human data, including informed consent, individual privacy, harm, and data re-identification.

The reputation of a research institution encourages individuals to participate in research (Conley and Pocs, 2018). Any unaddressed concerns or privacy incidents can severely affect participation, which voluntary research on data from human subjects relies heavily upon (Kelsey, 1981; Couper et al., 2008).

Inspired by Shneiderman, we argue for auditing, independent oversight, and trustworthy certification for data accesses. Building accountability around the applicable laws and the dynamic privacy risks landscape is the way forward (Kroll et al., 2019; McGraw and Petersen, 2020). Subject's perception of privacy might change over time and depend upon the purpose data is collected for (Sharma et al., 2020). Also, the interpretation of sensitive data is a topic of debate among technologists and lawyers. Data analysis techniques, such as *statistical inferences* can blur the lines between personally identifiable information (PII) and not-PII (Kroll et al., 2019).

In this paper, we present Lohpi: a safe and accountable data processing infrastructure for participatory research projects, designed specifically for medical and sport sciences that handle sensitive personal data. We argue for a decentralized approach where research institutions can process data on their infrastructures and maintain control of data assets, rather than a central one-fits-all service. Collaborations are facilitated using a resilient metadata distribution substrate, implemented using gossip-based data exchanges (Johansen et al., 2015b), which is a probabilistic data dissemination scheme. The key contribution of Lohpi is our compliant data analytic infrastructure that encapsulates and manages distributed data assets. Data usage policies reside as *meta-code* (Hurley and Johansen, 2014; Johansen et al., 2015a), stored at the file system level, along with the data they govern and updated via *gossips*. We present our initial results of propagating policy updates as gossips (6) with fault-tolerant behavior.

## 2. Background

**Figure 1:**
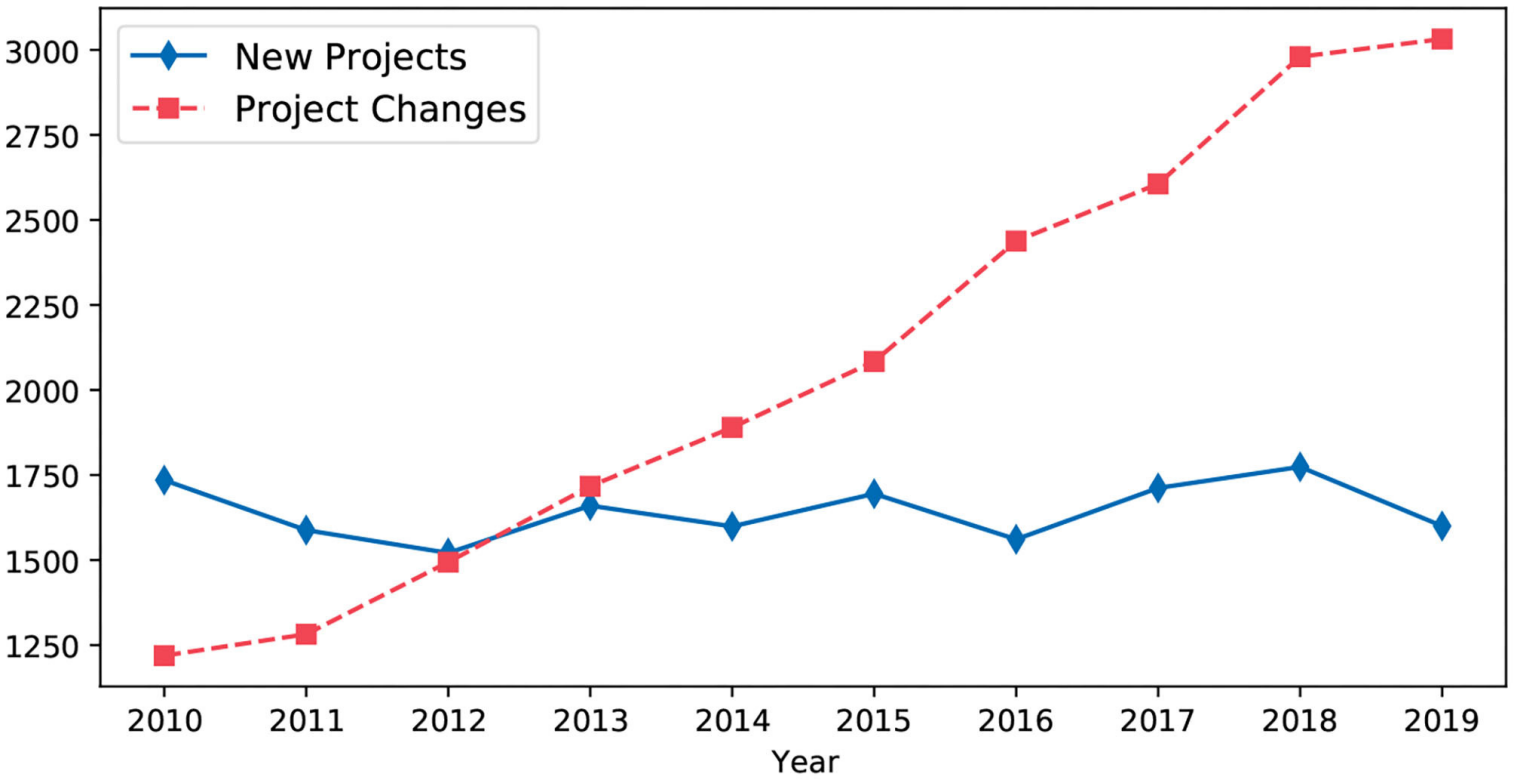
Different types of applications processed annually at Norwegian ethics committee (REK, 2020).

Salerno et al. discuss the ethics in computing in the context of big data, epidemiology, and public health. They discuss concerns where multiple data sources can be linked without a subject's *informed consent*, which can result in re-identification of the subject. Protecting data shared on a global scale is identified as one of the key challenges. Since healthcare research projects need an ethics committee approval, we collected data from annual reports of the Norwegian ethics committee (REK, 2020). Figure 1 shows the growing number of changes to existing projects. We contacted them over e-mail to clarify what constitutes as a *project change*. It includes changes to people who have access to the collected data (*new researchers*), newly discovered risks for subjects (*new threats*), and even changes in conditions for dispensation from professional secrecy requirements (*new laws*). These changes need to be approved by an ethics committee. To the best of our knowledge, we are not aware of mechanisms that an ethics committee can use to verify compliant data processing by researchers. Also, anonymized data available in repositories, such as Dataverse (King, 2007) can be re-identified and misused (Dāvida, 2020). We are building Lohpi as a platform for compliant data usage among researchers, which might identify a *rogue researcher* (Camden, 2005).

Data collected in research studies is often used beyond its initially specified goals (Cheung, 2018). We introduce one example from an epidemiological study known as the Tromsø Study (Thelle et al., 1976), which involves 10-fold thousands of subjects (Nilssen et al., 1990; Brækkan et al., 2010; Jacobsen et al., 2012). For such studies, statistical inferences can produce correlations that are novel. For instance, Svartberg et al. (2004) analyzed correlations between waist circumference and testosterone levels in men from the Tromsø Study data collected from 1994 to 1995. They found that the correlation with waist circumference was stronger compared to other indicators, such as BMI or waist-hip ratio. Similar novel correlations can be exploited for commercial interests (Dāvida, 2020). Kroll et al. (2019) argue that it is difficult to differentiate sensitive data. Perceived sensitivity of some data object can also change from the time it is collected to the time it is analyzed. For instance, knowledge of correlations as found by Svartberg et al. and its coverage in media can change the perception of subjects toward their privacy. Kroll et al. and Shneiderman argue for building oversight to better understand data usage and build a complete picture.

Lohpi is designed to promote collaboration among researchers, not limit it, by including accountability on research-data processing as a core feature. We conjecture that by building accountability into the system, we can improve the trust between researchers and the public. That may lead to improved participation in fields, such as epidemiology, which rely heavily on public participation (Couper et al., 2008).

### 2.1. Regulatory Requirements

Data-driven healthcare research requires scrutiny of and compliance with data protection laws, such as the European General Data Protection Regulation (GDPR). GDPR Article 89(1) provides important research exemptions, provided that adequate technical and organizational safeguards for the rights and freedoms of the data subject are put in place. And GDPR Article 4(7) stipulates that data controllers are obliged to determine the purposes and means of the processing of personal data, which can be subject to exceptions in Article 14(5)(b). Also, the data protection instrument allows for *broad consent* only in specific research areas and subject to recognized ethical standards in Recital 33 of the GDPR. The exact implications of GDPR on research is still a subject of academic debate (Norval and Henderson, 2020). Data-driven healthcare research raises both legal and ethical questions to maintain data subjects' rights (van Veen, 2018).

The exemptions provided in GDPR for research projects entrust the responsibility on ethics committees. An ethics committee, also known as Research Ethics Committee (REC), Institutional Review Board (IRB), or Data Protection Officer (DPO), is charged with the task of checking that a research project complies with applicable laws, regulations, and ethical standards. Therefore, throughout this paper, we show the functions of Lohpi in context of an ethics committee concerning research data processing. In this paper, we assume that an ethics committee safeguards the privacy of individuals that contribute personal data to a research project. Therefore, the ethics committee provides updates for an existing project in Lohpi, either as consent changes or data-access policy changes.

### 2.2. FAIR and FAIR-Health

The FAIR Guiding Principles (Wilkinson et al., 2016) are becoming an established standard for research data. The principles can be applied to data assets to make them Findable, Accessible, Interoperable, and Reusable. Wilkinson et al. provides a detailed guideline on how these principles can be applied to data and non-data assets. Holub et al. (2018) proposed an extension to the FAIR data management principles by accounting for the privacy risks associated with research data. Their work maps the flow of data from participants to research data repositories and highlights the trust and privacy aspects. Their work considers a research project compliant if the consents are obtained either by the participants or an ethics committee. They highlight the competing interests in human data use in research. These are (a) protection of privacy of individuals (b) reuse of data, and (c) complex ownership and economic interests. Anonymization cannot always protect individuals' privacy in shared data (Holub et al., 2018).

Holub et al. use GDPR Article 9 as a reference for sensitivity of data. They introduce additional requirements on data sharing as part of FAIR-Health principles. They advocate for checking compliance to research data before it is shared. They do not discuss who is responsible for compliance when sharing data. Lohpi allows checking for compliance with approved policies at any stage of a project that can be initiated by an ethics committee. The purpose of auditing is to discover non-compliant behavior, its cause, and a possible fix. Additionally, audit reports can be useful for subjects to understand what their data is being used for and by whom. Providing control over one's data is crucial for building trust (Hoffman et al., 1999). Kroll et al. argue for a global view of data usage that puts a Research Ethics Committee (REC) in a better position to evaluate privacy risks and mitigate them. Additionally, having such auditing mechanisms can be used to evaluate and verify the *ethical* contracts between the subjects and researchers (Lynch, 2011). Therefore a non-compliant data use by a researcher can be detected and avoided before it causes any serious privacy harm.

Interoperability and collaboration are often required in large projects where collaborators join at a later stage (Conley and Pocs, 2018). Often subjects are not aware of these future collaborators. In line with the WHO's definition, an ethics committee must protect the subjects from any harm (World Health Organization, 2009). As argued earlier, an ethics committee is in a better position to have a global view of data usage by various researchers. Since Lohpi supports dynamic policies, an ethics committee can issue policy changes, which reflects a *consent*, changes to existing consent, or even revocation of an existing consent. Additionally, newly enforced laws or recommendations from a Data Protection Agency for sensitive data can be enforced by the ethics committee. Lohpi can ensure enforcement of up-to-date data-access policies which are designed to protect against newly discovered vulnerabilities.

## 3. Lohpi Overview

In Lohpi, one or more *subjects* contribute data for use in one or more research projects. Researchers analyze this data in the context of research projects. The output is to be used for the *common good*, outside the constraint of the project, for instance in a public medical journal.

Each project is owned and hosted by a single research institution, but might be accessed by researchers across multiple institutions. The research data is hosted on storage nodes owned by institutions. These storage nodes form a data storage network in Lohpi. Lohpi does not use a centralized storage or processing, so researchers can work on and process data on their own computing infrastructure. This is a key design principle, as centralized processing does not scale well with the increasing complexity in big-data analytics methods.

**Figure 2:**
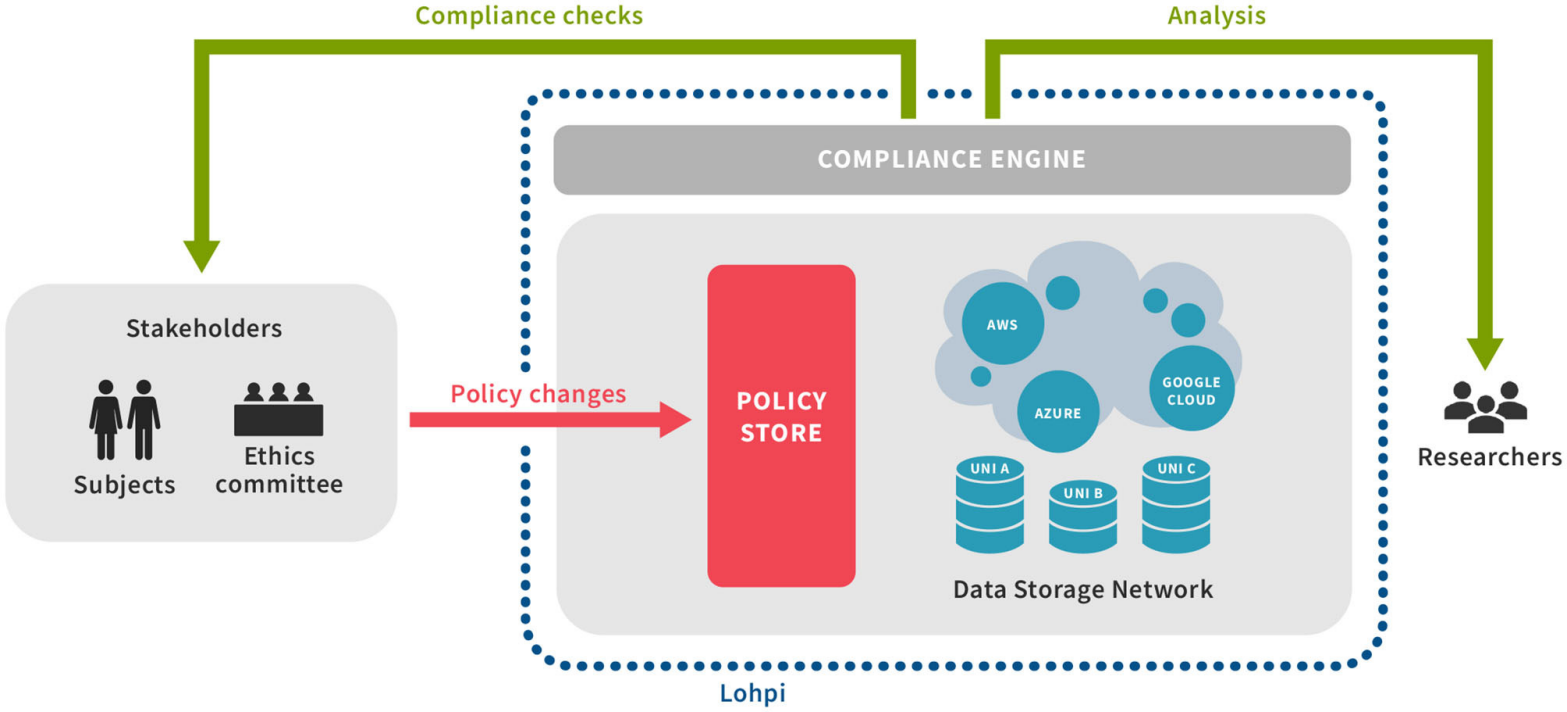
Overview of Lohpi and interaction with Ethics committee and Researchers.

The primary function of Lohpi is to manage, distribute, and coordinate metadata related to access control and data usage, and to enforce data usage policies based on that metadata. Participating institutions host one or more nodes in the Lohpi network: a peer-to-peer like substrate where members collaborate to disseminate up-to-date metadata about the projects, datasets, data usages, and subjects' consents to participation in various projects. Compliance with law, regulations, and subjects' wishes is enforced by a combination of data provenance, file system monitoring, auditing, and container isolation. This data-processing model is summarized in Figure 2.

The research data is stored in a federated manner at multiple nodes forming a data storage network (3.4). These nodes and other key components, such as policy store and directory server communicate over a secure protocol. The security aspects are discussed later in this section. Data-access policies that may arise from ethics committees or subjects themselves as consent, reside at the policy store. The policies and any changes to them are handled by the policy store (3.2). The policy store disseminates these changes through gossips. Any egress of data is checked against the applicable policy and may get logged at various components. These generated logs are used by the compliance engine (3.5) to determine compliant behavior. An ethics committee can also request compliance checks on data-accesses, projects, or nodes.

Lohpi is not intended to replace existing data collection and storage infrastructure and tools. Instead, Lohpi integrates and federates existing data hosting services to implement global access policies for datasets and data consumers across institutional and administrative domains. These nodes are owned and administered by different institutions that act as the data providers. They communicate over the secure Transport Layer Security (TLS) protocol with each other (3.1). As such, Lohpi enables compliant data use in the age of open-data access and collaboration among researchers and interested third-parties.

### 3.1. Security Aspects

Lohpi is intended to operate as a permissioned peer-to-peer-like system for research organizations that want to cooperate on sharing a potential large portfolio of research projects and datasets. Storage Nodes or simply *nodes* that are to participate in the network are admitted by possessing a valid X.509 certificate, signed by a trusted Certificate Authority (CA). Secure and mutually authenticated communication channels are established between nodes using the TLS protocol in combination with the obtained X.509 certificates. A correct node will reject connection attempts from nodes that do not have a valid X.509 certificate signed by a trusted CA.

Lohpi relies on gossips to exchange updates between its member nodes. Gossip protocols provide robust scalable communication for a large-scale distributed system (Vogels et al., 2003). The other advantages of gossip protocols are bounded load on participants, topology independence, convergent consistency, and simplicity (Birman, 2007). The gossip messages are issued by the policy store (see 3.2), which signs them for authenticity. Upon receiving an update, either directly from the policy store or other nodes, messages are cryptographically verified before any further processing can take place. The underlying *Fireflies network* (Johansen et al., 2015b) maintains membership in a verifiable structure that is resilient toward intruder nodes. The Fireflies overlay manages the membership of nodes for our large gossip network.

To authenticate users, we integrated Lohpi with existing authentication services, such as OpenID (Recordon and Reed, 2006). We assume that institutions can verify the identity of their affiliated researchers through their existing authentication framework. Researchers accessing the system must be affiliated with a participating institution already known to Lohpi. In our current implementation of Lohpi, we rely on OpenID for institutional authenticated identities. We use JSON Web Tokens (JWTs) to transfer authentication claims between parties.

### 3.2. Policy Store

The policy store provides mechanisms to update data-access policies inside Lohpi. As seen in Figure 2, an ethics committee can issue policy changes. These changes may reflect various changes, such as consent withdrawal, added collaborators, etc. Policy changes are then verified for authorization based on the issuer and disseminated as gossips (see 4.1). Each policy is version controlled in a *git*-like fashion and each change to a policy has additional details for enhanced accountability. These details are stored and periodically backed up for failure-recovery.

The core functions of the policy store include identifying and issuing policy updates. An ethics committee can view research projects and associated policies. Through APIs the policy store facilitates viewing and changing policies. These APIs can also be used to evaluate policies using a third-party or the compliance engine. When a committee issues changes to a policy, the policy store uses the last state of the policy and processes changes to issue relevant updates. It might happen that a change does not issue a policy update if the existing policy is broad enough to allow such access. All updates issued by the policy store have unique identifiers, issuer's reference, and timestamps. They are digitally signed by the policy store. The signing allows the data storage nodes to verify the authenticity of updates.

### 3.3. Directory Server

**Figure 3:**
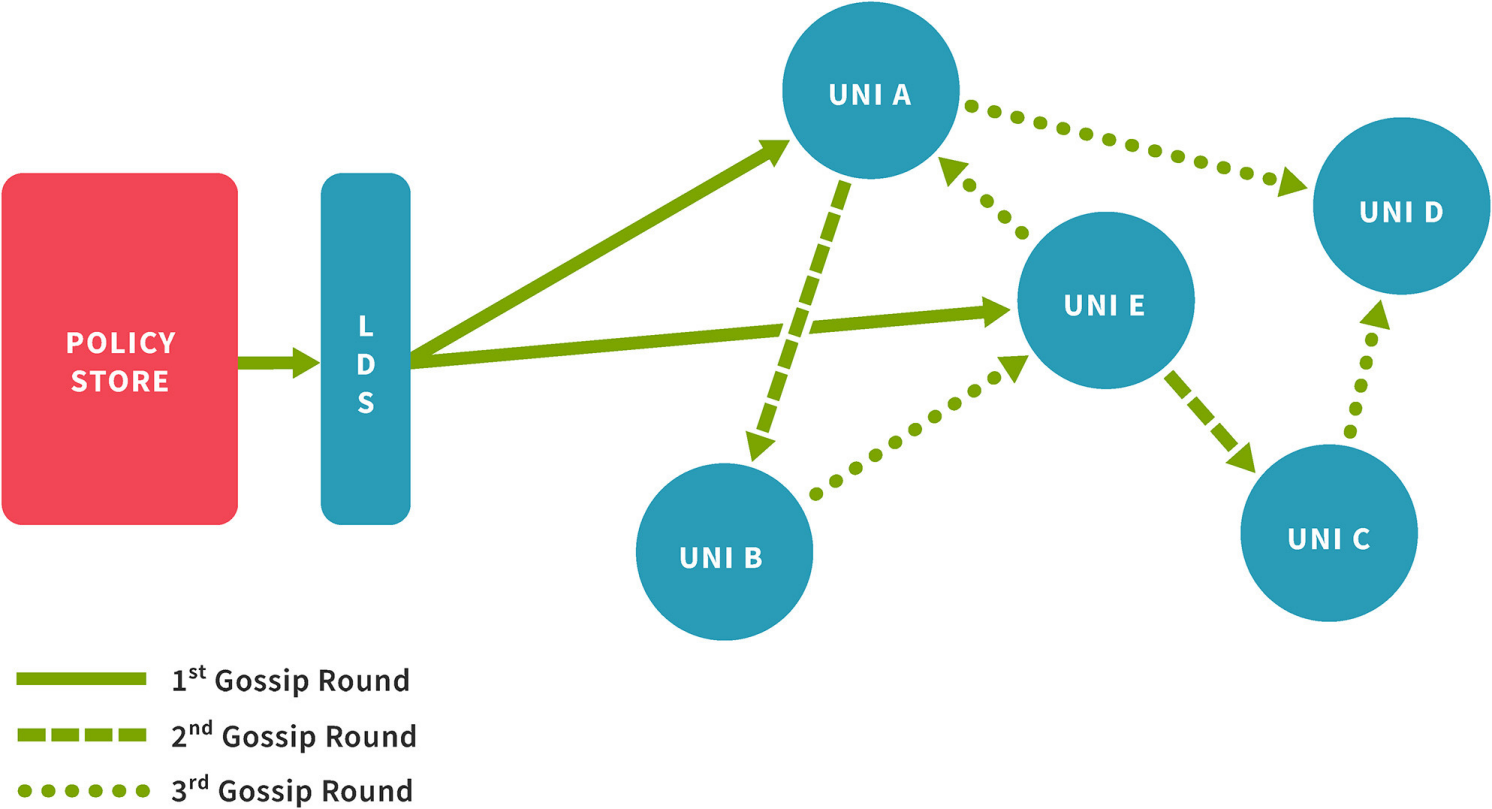
Disseminating a policy update as a gossip in Lohpi.

The Lohpi Directory Server (LDS) interfaces between the policy store and data storage network (see Figure 3) and is a key element of the underlying Fireflies network. The directory server maintains an in-memory collection of the nodes' references, which are used for different functions, such as *gossiping* (see 4.1), *probing* (see 4.2), etc. The directory server can collect metrics for system's state and performance evaluation.

The directory server is one of the first components to boot up when starting a Lohpi network. Once it is online, storage nodes can join the network. When a new storage node joins the network, it performs a handshake to exchange message authentication keys with the directory server. A node must present a valid certificate to join the network. It exchanges meta-information about itself and the research data it stores with the directory server.

### 3.4. Data Storage Network

The data storage network comprises of multiple *nodes* that store and process data in compliance with recent data-access policies. Lohpi is designed to be agnostic about data format, and therefore handles datasets as opaque objects. A metadata file is supplied along with the data to identify different data types and file structures. The metadata is stored to facilitate analytical processing, identifying sensitive data, and compliance queries by an ethics committee. A node is owned and maintained by a research institution. A contact email address is required for receiving operational emails associated with a node, in case of failures or compliance issues. A node needs to be aware of only the CA, directory server, and the policy store to join the network. The set of data storage nodes in Lohpi is represented by *N*.

Each node is also part of the underlying Fireflies network (Johansen et al., 2015b). As per the design of Fireflies, each node gossips a received gossip message to its neighbors (see 4.1). Every gossip may not contain relevant updates for a node. Upon receiving the updates, we classify parts of updates as relevant or not. The relevant are the ones that are applicable to the data stored at a node. The irrelevant updates are not applied, however, their object identifiers and version number are stored in a local table for lookup later. A node can share the observed information about received gossip messages with the compliance engine. They can then be utilized by the compliance engine along with other information to detect and identify the source of compliance failures.

### 3.5. Compliance Engine

The compliance engine facilitates compliance checks in the Lohpi system. Compliance can be requested or configured automatically in Lohpi. For each data egress query, the corresponding node stores immutable logs about the current state of policies available at the node. A node verifies a data-request based on the policies available at the node. There might be cases where the current data-access policies are available at the node or a misconfiguration has lead to unwarranted access. During a compliance check, the compliance engine asks for the state of various objects, such as policy information or recently seen gossips from a node. The compliance engine also collects such information from one or more neighbors of a node. The neighbors are randomly selected to avoid any collusion. The compliance engine then determines based on the information if the node's behavior is compliant or not. There can be communication issues in the system's gossip network that can be identified by these checks. Upon investigation, concerned parties can be notified along with remedial actions. The compliance engine can also send reports to the node owner. Subsequent actions can then be taken by the node administrator to fix trivial issues, which may include re-syncing policies directly from the policy store. Such methods are supported by the policy store but are designed to use very rarely to recover from stale policies. Lohpi can be configured to perform such compliance checks periodically.

Alternatively, an ethics committee can investigate data-processing on a research project to find non-compliant behaviors. With Lohpi, an ethics committee can define custom-compliance checks and run them against the research projects that it has approved. We argue that through regular compliance checks, an ethics committee can identify and mitigate privacy risks that may exist in some projects.

## 4. Communication Substrate

Lohpi nodes collaborate to provide a secure and reliable communication substrate. In this section we describe the two core functions of this substrate: update dissemination and probing. A policy change is disseminated by the policy store as a gossip message. The nodes in the data storage network (3.4) then gossip the message among themselves. As nodes join and leave the network, the dissemination of gossips may not be optimal. Probing is used to tweak the performance of the data storage network. The protocol is described later in this section.

### 4.1. Update Dissemination

We assume that multiple nodes in the data storage network (3.4) have stored data along with their policies. A policy change has been approved by the ethics committee that limits sharing of medical data stored at one of the nodes. The policy change is propagated to the target node using *gossips* in the network. Upon receiving a policy change, the policy store validates the authenticity of the issuer (ethics committee). An ethics committee can be responsible for handling many projects and subjects. The policy store compiles these changes in a two-part gossip message. The first part contains meta-information about the update, in the form of a map of object identifiers and their version numbers. This map identifies objects that are affected by the updates contained in the message. At a node, an approach similar to Sharma (2016) is used to determine if the update is relevant or not. The second part of the message contains the complete policy for objects contained in the first part. This includes more information about the update, such as date, issuer, and reference. After preparing a message, each gossip message is signed by the policy store to protect the integrity of the system. Additional details, such as message identifier and timestamp are also added before sending the message to the data storage network.

When at least ϕ percent of the data storage network receives a message sent by the policy store, we consider it successful. Once a gossip message has been prepared, the policy store multicasts the message to a set of nodes denoted by *S*.

(1) $$\begin{array}{r} S\subset N \end{array}$$

By multicasting the same message to a subset of the network, the system can reach the threshold ϕ faster without creating a significant overhead at the policy store. After the initial multicast to *S*, the policy store relies on the nodes to gossip the message throughout the network. The nodes in *S*, begin *gossiping* the message to their neighbors and then their neighbors and so on (see Figure 3). This process keeps on until a new gossip message is introduced to the system. If no new gossip message is introduced to the system, the system continues to gossip at fixed intervals. The nodes ignore duplicate messages by using message identifiers. The number of nodes in *S* is represented by σ i.e.,

(2) $$\begin{array}{r} \sigma =|S| \end{array}$$

Gossiping depends largely on the pseudo-random network topology (Johansen et al., 2015b). We set the goal to reach ϕ percent of the data storage network in τ time. τ can also be seen as the time interval between two subsequent gossip messages introduced by the policy store. A large value of τ will reduce the number of gossip messages that can be introduced. We have additionally built a *probing* mechanism in the Lohpi network, which can periodically fine-tune the parameters σ and τ. We consider the parameter ϕ to be defined by system administrators and not be changed frequently. Additionally, we apply an upper limit for σ (see 5) to prevent a bottleneck at the policy store and offload the message passing to the data storage network as gossips.

### 4.2. Probing Protocol

Distributed systems can have unpredictable behavior due to node failures and transient network outages. We therefore include a probing and recovery mechanism that detects and mitigates such problems. The performance tuning parameters described above allow Lohpi to batch policy updates efficiently while reaching the predefined acceptable coverage among the nodes. We will next explain the probing mechanism.

Let ϕ be the percent of the data storage network that must receive a gossip message to consider it successful. The value is in the range

(3) $$\begin{array}{r} 0<\phi \leq 1 \end{array}$$

In gossip-based systems, it can be challenging for a gossip to reach all nodes in a limited time. Instead of waiting for all the nodes to acknowledge, we wait for a threshold value derived from ϕ and *N*. We call it *k* and it is calculated as

(4) $$\begin{array}{r} k=\max\left(\left\lceil \phi \times |N|\right\rceil ,\phi \times |N|+1\right) \end{array}$$

τ represents the time interval between two messages sent by the policy store, typically in seconds. σ represents the number of nodes to which the policy store multicasts the update directly. For example, if the policy store multicasts the message to one node, σ = 1.

In a default configuration, σ starts with value 1. The policy store creates and marks gossips as *probing messages*, such that the nodes acknowledge back to the policy store. In a typical manner, the gossip message is composed and sent (see 4.1). The policy store gossips the probe message and waits for at least *k* acknowledgments from unique nodes. Duplicate acknowledgments are handled by the policy store. After τ seconds have passed and the threshold is not met, the system assumes that the probing has failed. This causes the system to assume that the current configuration is not optimal. To overcome this, the system increases the value of σ by an order of 2. After that, the policy store starts a new probing run. The new probing run is highlighted in the messsageID field, which is returned in the acknowledgment by the nodes. Then the policy store selects the remaining number of nodes required to match the increased value of σ. The selection of nodes can be random or based on algorithms, such as Least Recently Used (LRU), depending on the configuration. After selecting, the policy store starts over and multicasts a new probe message to these nodes. Then, the policy store waits for τ time to receive at least *k* acknowledgments. This continues until σ reaches the limit, which is defined as

(5) $$\begin{array}{r} \sigma \leq \frac{\phi \times |N|}{2} \end{array}$$

We define this arbitrary limit to maximize the utilization of gossiping within the network. We argue that a high value of σ can lead to a bottleneck at the policy store. Once this limit for σ is reached, the system begins increasing the value of τ. The values are only modified if the acceptable number of nodes are not reached in the given time limit. Like previously, the system begins probing again with modified τ and this continues until three consecutive probing requests are successful.

After three successful probing requests, the new parameters are applied to the system. The new values for σ and τ are logged and subsequent gossips by the policy store use them. In case the system cannot obtain a configuration even after multiple attempts, the value of ϕ may need to be changed. For example, if ϕ≈1, it might be difficult to find appropriate values of σ and τ. If the system fails to obtain a configuration within limits through probing, it generates an alert message for the administration. The results of probing requests are logged regardless. Probing may lead to an intervention by a system administrator, if necessary.

## 5. Evaluation

A key property of Lohpi is reliable dissemination of policy updates. We therefore evaluate the propagation of the updates as gossips, issued by an ethics committee and introduced to Lohpi by the policy store. Jenkins et al. (2001) evaluated gossip-based propagation and provided a mathematical model for message dissemination. Their model calculates dissemination probability based on number of gossip-rounds. Unlike Jenkins et al., we measure the time (in seconds) for propagating an update. We evaluate multiple scenarios, which are detailed in 5.1. Later in 5.2, we describe our evaluation of the access controls' overhead of reading operations.

### 5.1. Update Dissemination

As described earlier, Lohpi facilitates updating a project's data-access policy. We designed a set of micro-benchmarks to evaluate the time it takes to propagate a data-access policy change. These experiments evaluate the time required to propagate an update under different conditions. Additionally, we demonstrate the fault-tolerance of the system, by introducing synthetic node-failures in the system.

We begin by simulating the growth of the total number of nodes *N* in Lohpi. We define static values for ϕ and begin introducing updates in the system using the policy store. Similar to *probing* (4.2), we configure the data storage network to acknowledge receiving an update. Duplicate acknowledgments are ignored by the policy store. To consider a policy update successful, the policy store must receive *k* acknowledgments (see 4). We measure the time elapsed after the policy store multicasts the message to σ set of nodes and waits to receive *k* acknowledgments. We arbitrarily chose the message size to 512 KB. We take measurements at least three times to calculate uncertainty and plot them using error bars. After recording the first set of readings, we increase the value of σ, by doubling it and take a further set of readings. Note that the upper limit of σ is bounded by Equation (5). Since the value of ϕ is static, we evaluated the whole set of experiments again with a higher value of ϕ.

We evaluate the fault-tolerant nature of Lohpi as well. As discussed in 3.4, a data storage node is run and maintained by an institution instead of a centralized server. Many conditions, such as network failure, power loss, or natural calamities can cause a node to fail or simply appear offline to the network. By fail, we imply that a node is not able to receive and/or gossip an update further into the data storage network. Like earlier, we measure the propagation time of a message and compare it with a network without failures. We used the same values for *N* as earlier and simulate different sizes of the network. We induce failure in the network, in terms of ϵ percentage of nodes. The possible values for ϵ are

(6) $$\begin{array}{r} 0\leq \epsilon \leq 1 \end{array}$$

where ϵ = 0 means no failures and ϵ = 1 means all the nodes in the data storage network have failed. After introducing a controlled amount of failures, we evaluate the effect of increasing σ to mitigate the performance issues that might be induced by failed nodes. We used an Intel Xeon E3-2370 cpu^1^-based blade server with 64 GB memory for running our simulations. We vary the network size (*N*) from 2 until 64, doubling it in every step. Other values, such as ϕ, σ, and ϵ are varied as well and discussed in 6.

### 5.2. Overhead

We also evaluate the overhead added by the access controls. First, we measure the *baseline* by reading multiple files from the file system without any access controls introduced by Lohpi. After enabling the access controls, we perform the same read operation and measure times. We measure the time required to read a large chunk (1 GB) of data. We have designed Lohpi to facilitate different studies which may have different data file sizes. Additionally, a subject's data might be reflected as one row in a large study's data. Breaking down a study's data into multiple files which reflect individual subjects can lead to fine-grained controls that might add additional overhead. We evaluate this by varying the file sizes while keeping the total read data to be constant. For example, if each file is of size 4 KB, there will be 262,144 files to read for ~1 GB of data. We present the results and discuss them in 6.2.

## 6. Results

We examine the results obtained from our simulation of the Lohpi network. We first focus on the propagation of messages through the distributed storage network. Later in this section, we present the overhead added by the access controls.

### 6.1. Update Dissemination

**Figure 4:**
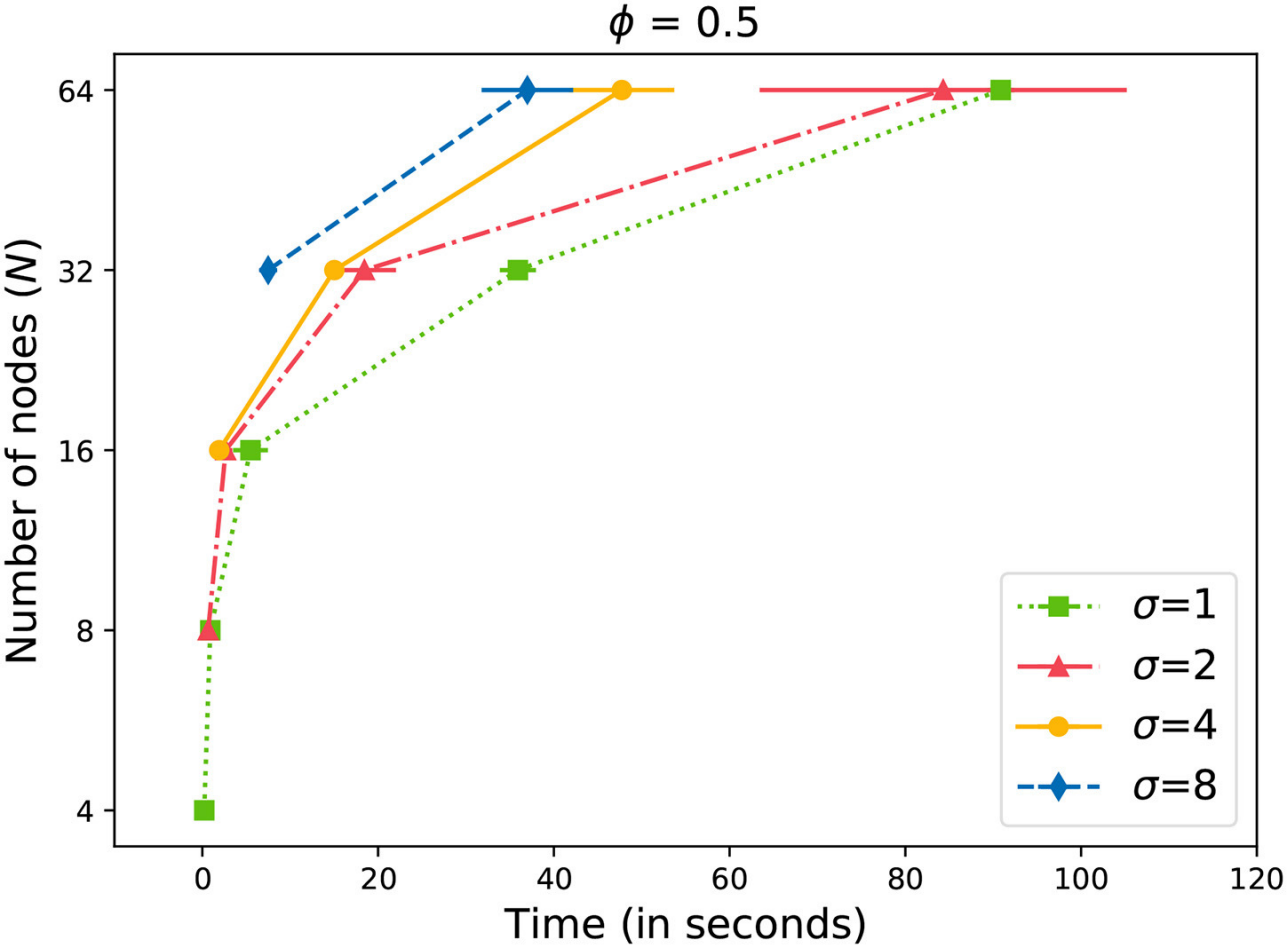
Time to gossip a message with ϕ = 0.5.

Earlier in Equation (4), we described the criteria to consider a message successfully propagated through the network. In Figure 4, we observe the time required for reaching at least half of the data storage network. We can observe that the time required to reach the acceptance level grows exponentially with the number of nodes (*N*) in the network. We also observe that by increasing the value of σ, we can propagate the message faster through the network. However, the gains are not significant at lower values of *N*. Only with *N*≥ 32, we start to observe significant gains. Also, the variability in the time increases with the size of the network.

**Figure 5:**
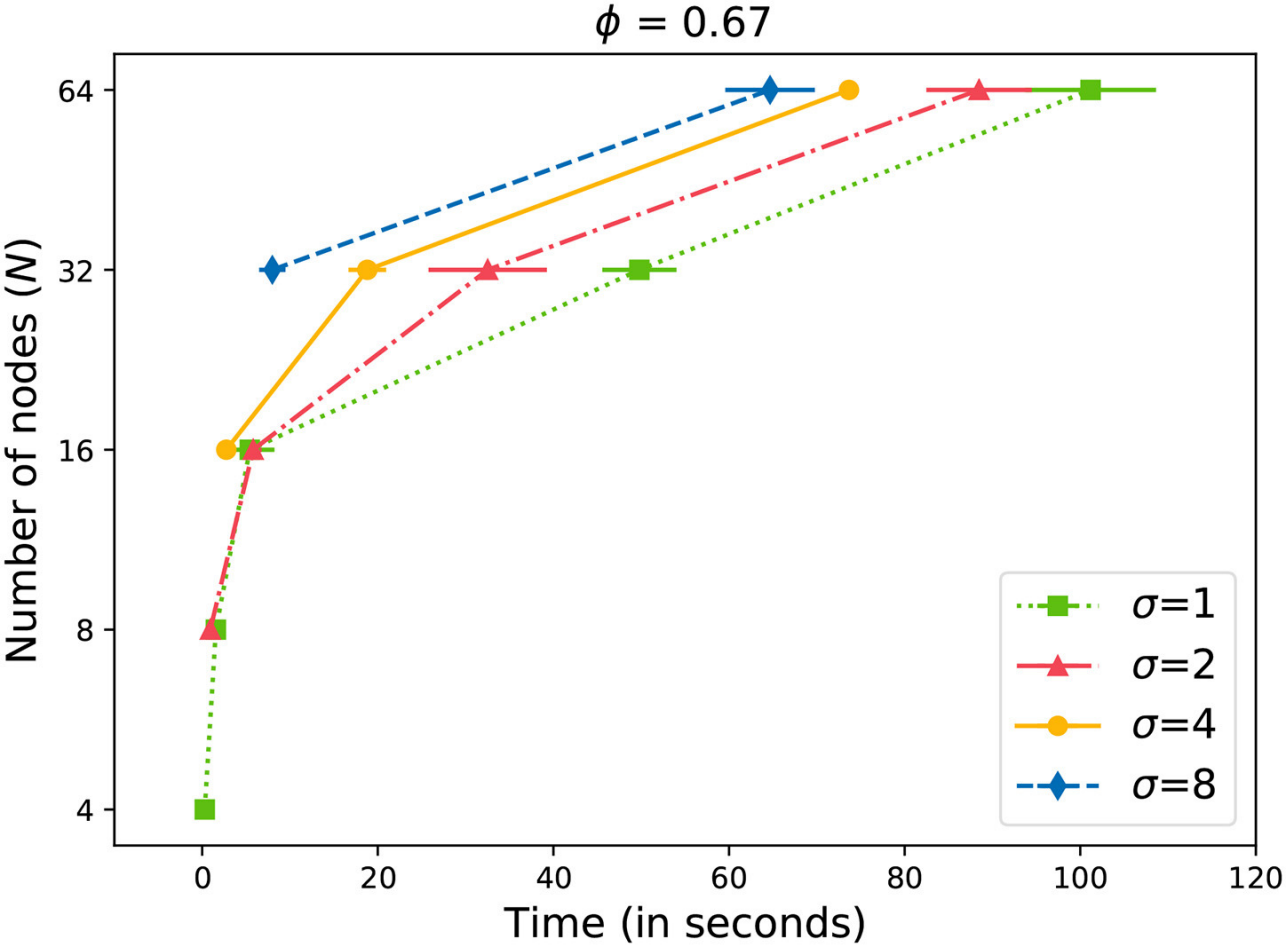
Time to gossip a message with ϕ = 0.67.

Similar to Figure 4, we can observe similar behavior in Figure 5 with a slightly higher value of ϕ = 0.67. Interestingly enough, the differences are also significant at *N*≥ 32. Compared to Figure 4, we observe that more time is required to reach the acceptance level with a higher ϕ value in Figure 5. The differences increase as the network becomes larger.

**Figure 6:**
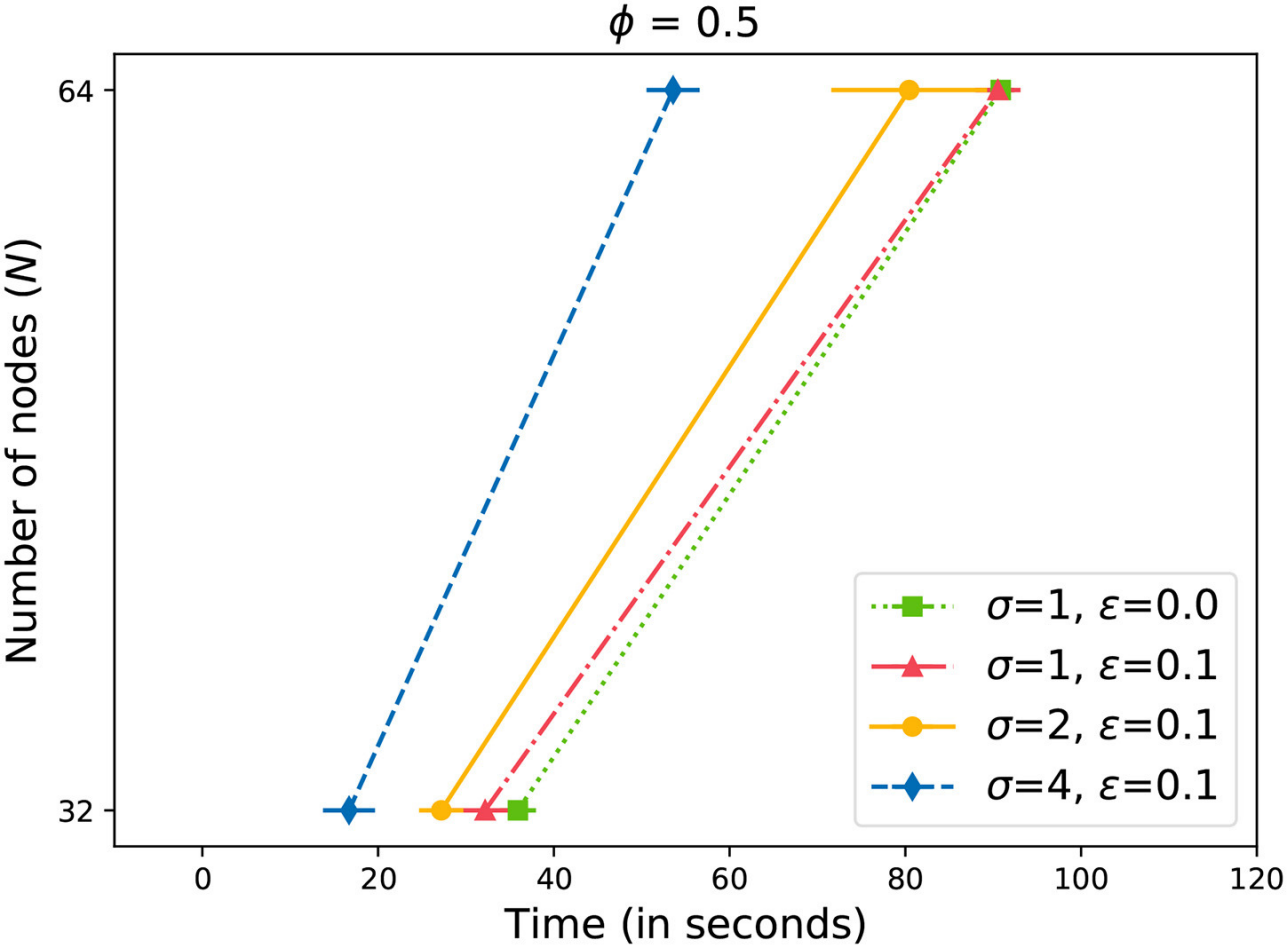
Time to gossip a message with 10% failed nodes.

**Figure 7:**
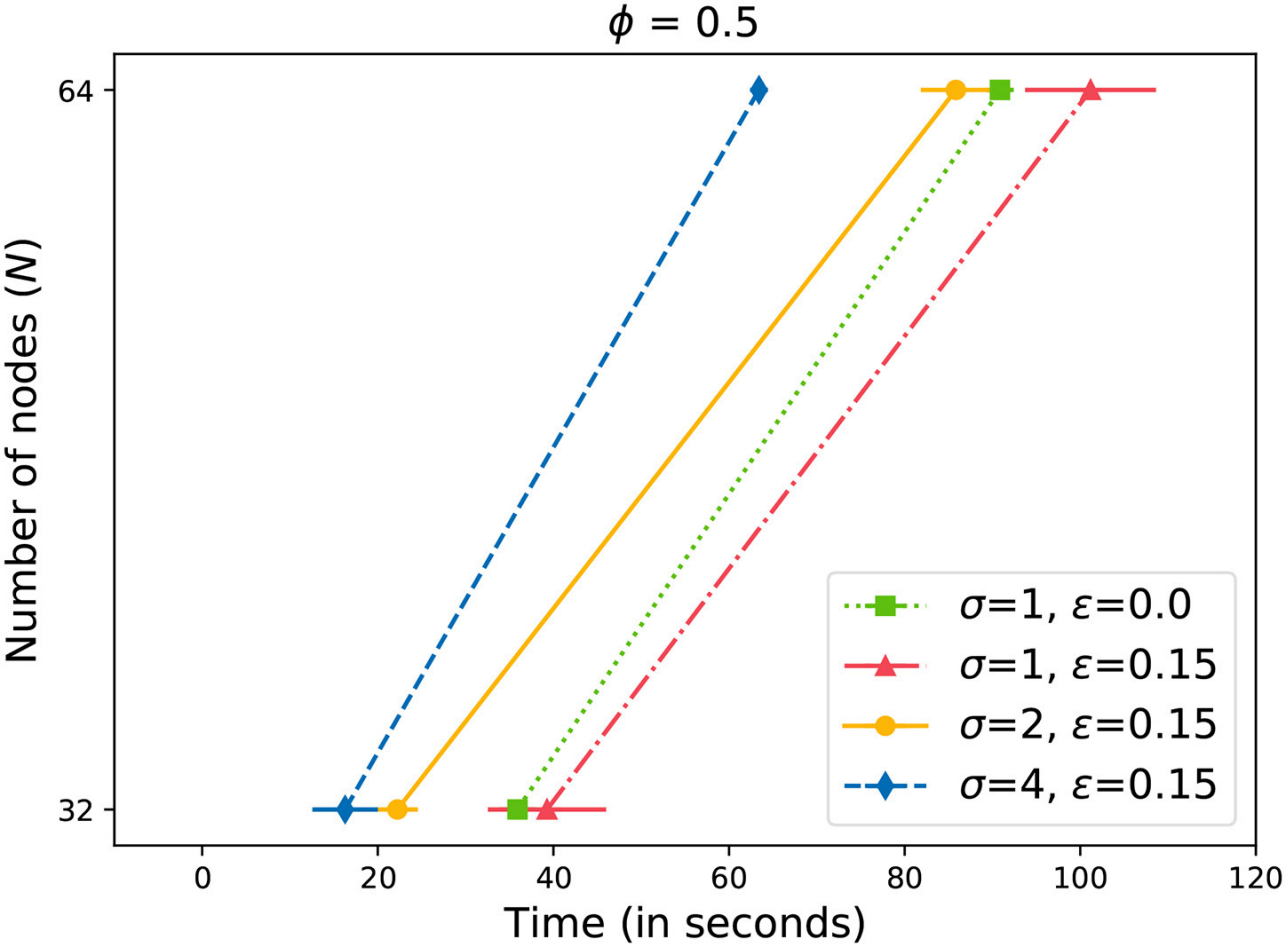
Time to gossip a message with 15% failed nodes.

**Figure 8:**
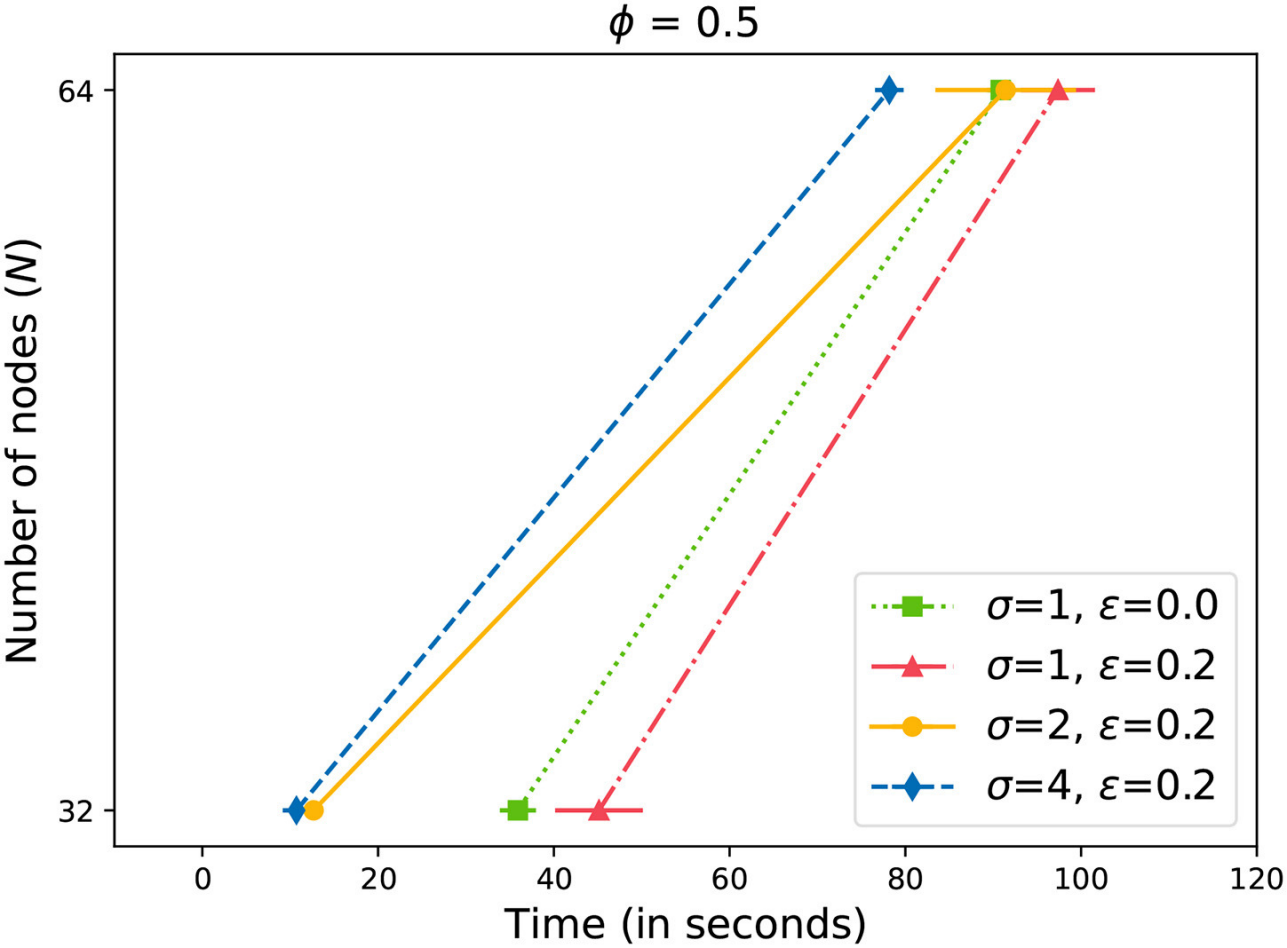
Time to gossip a message with 20% failed nodes.

Next, we present our results with induced failures in the network. Figures 6–8 plot the time to receive *k* acknowledgments while nodes in the system have failed. We can observe that in the case of 10% failures, the system corrects itself by re-forming rings (Johansen et al., 2015b). The performance is comparable to a system with no failures. As a result, the system does not cross the baseline where ϵ = 0 (Figure 6). Only with higher values of ϵ≥0.15 (Figures 7, 8), we can observe a drift towards the right, indicating more time required for a message to propagate with failed nodes. It is important to note here that we did not lower the value of *k* (4) while measuring the time with ϵ failures. For example, let us assume that *N* = 64, ϕ = 0.5, and ϵ = 0.2. From Equation (4), we can calculate *k* = 33. Even with 13 failed nodes, resulting in *N*′ = 51, we measured the time to receive 33 acknowledgments. It is evident from Figures 7, 8 that by increasing the value of σ, we can overcome the additional delay in propagating changes caused by nodes' failure. However, we need to keep in mind that there is an upper limit for σ (5). In our experiments, we doubled the value of σ in each step. The results illustrate that the updates can be propagated within an expected time frame.

### 6.2. File Access Overhead

**Figure 9:**
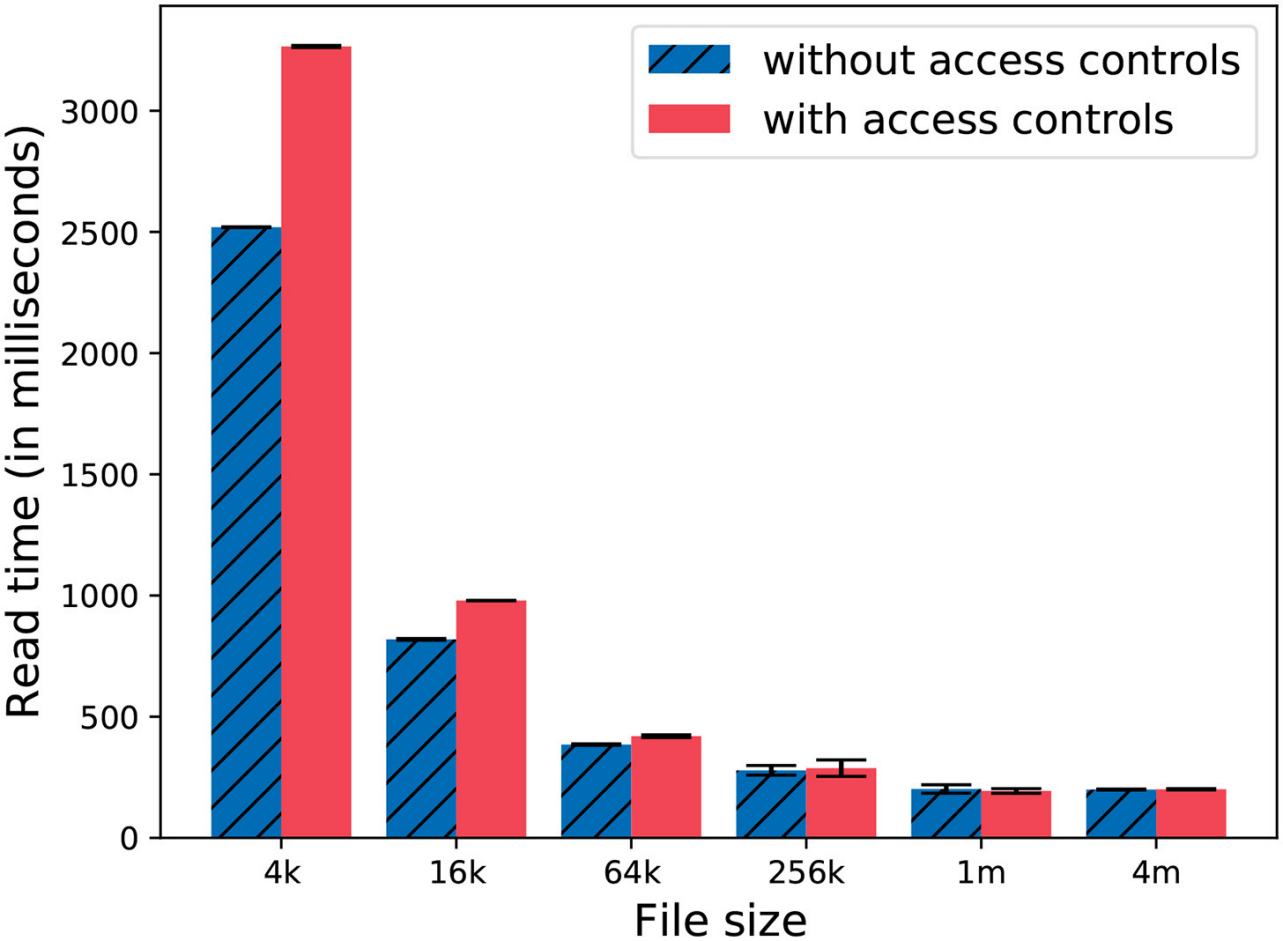
Reading 1 GB of data with different file sizes.

We added access controls based on the attributes of the researchers. These attributes may reflect association, country, or research groups. We evaluated the overhead added by our access controls for a file read operation by reading a large chunk of data. We repeated the experiment to observe the effect of file sizes on the overhead as well. The results are shown in Figure 9. We can report that the overhead is significantly large (≥15%) when the file sizes are smaller than 64 KB. As the file size grows, the overhead becomes much smaller. At file size = 4 MB, the overhead is almost negligible. We can argue that if study data is made available as a large archive in one file, the overhead for access control will be negligible. If the research data is made available as many small files, with each having its own fine-grained data access policy (reflecting each subject's preferences), the overhead can be significant.

## 7. Discussion

As shown in the results (6), our proof-of-concept demonstrates that it is possible to propagate updated policies promptly. We conjecture that within a larger distributed storage network, policy changes can be propagated within minutes. We demonstrated that the system can sustain multiple node failures. The probing mechanism can periodically tweak the network. We also conjecture that transparency in research data processing can increase public trust in research institutions and their projects involving real end-users. Adapting protection mechanisms to newly discovered threats to protect individuals in research can help sustain public trust (Mastroianni, 2008). Lohpi allows institutions to join the network and integrate with their identity management.

Making research data available only through Lohpi may seem against the FAIR principles (Wilkinson et al., 2016). Lohpi refers to the FAIR-Health principles (Holub et al., 2018), which consider privacy risks in research data. Lohpi facilitates compliant data sharing and analytics on research data involving humans, which may be from the fields of medical or sport sciences. Approaches similar to Meyer et al. (2012) might suit one large epidemiology project, such as the Tromsø, Study (Thelle et al., 1976), which has been running since the 1970s. However, such approaches are tied to project goals, which may change over time (Figure 1). Project changes may result in changes to data access policies. With Lohpi, we conjecture that an ethics committee can enforce changes to data access policies quickly and address concerns regarding data accountability.

Istvan et al. (2020) argue that GDPR support must be built at hardware-level with software-defined abstractions. In-storage computation using Trusted Execution Environments (TEEs) can enforce policies close to the data (Istvan et al., 2020). While Lohpi's approach is not centralized, we conjecture that the compliance engine can provide abstractions for an ethics committee to encode different compliance checks and run them on a data storage network. An expressive policy language like Guardat (Vahldiek-Oberwagner et al., 2015) can be realized using *meta-code* (Johansen et al., 2015a). We are also interested in building a tool to express research data usage protocol for streamlining REC approvals and verifying compliance against an approved protocol. We are interested in making existing research data in Norway available on Lohpi at multiple institutions.

## 8. Related Work

Many research institutions use Dataverse (King, 2007) to host and share research data. Dataverse is a centralized repository where researchers can deposit their data. By default, a dataset added to Dataverse has CC0 license. Researchers can add custom licenses. Users can accept such click-through licenses and access research data. Once a dataset is downloaded from Dataverse, there are no mechanisms to restrict sharing through any other means, such as over FTP or a USB-drive. Woolley et al. (2018) introduced the Automatable Discovery and Access Matrix (ADA-M) that allows stakeholders to confidently track, manage, and interpret applicable legal and ethical requirements. The ADA-M metadata profiles allow an ethics committee to evaluate and approve information models linked to a dataset. ADA-M facilitates responsible sharing outlined in the profile and allows the custodian to check the accesses against regulatory parameters. However, they do not mention any functionality about issuing updates to the profile. Alter et al. (2019) presented Data Tags Suite (DATS), which can be used to describe data access, use conditions, and consent information. DATS provides a metadata exchange format without any compliance checking mechanisms. Havelange et al. (2019) present their preliminary work that uses a blockchain-based smart contract to attach license requirements to a dataset. The datasets are encrypted and ADA-M profiles are attached with each dataset. A researcher accepts the contract and receives a token to decrypt the dataset. The researcher's data accesses are checked against the profile for compliance. They require each researcher, dataset provider, and a supervisory authority to have a node on the Ethereum-blockchain network. They do not provide any evaluation in their work.

## 9. Conclusion

We have presented Lohpi, a proof-of-concept distributed infrastructure to support compliant data sharing and analytics on research data. By leveraging a secure and scalable gossiping network (Johansen et al., 2015b), we ensure that the policy changes propagate in the network with minimum overhead at the policy store. The Lohpi architecture allows us to scale the policy store horizontally. When a research project spans multiple ethics committees, each ethics committee can have their own trusted policy store that enables a distributed set of data storage nodes to work together. As per our design, data storage nodes can run in the cloud or on campus hardware which allows different research institutions to join the Lohpi network without moving their data.

## Data Availability Statement

The original contributions presented in the study are included in the article/supplementary material, further inquiries can be directed to the corresponding author/s.

## Author Contributions

All authors listed have made a substantial, direct and intellectual contribution to the work, and approved it for publication.

## Conflict of Interest

The authors declare that the research was conducted in the absence of any commercial or financial relationships that could be construed as a potential conflict of interest.

## Footnotes

^1^https://ark.intel.com/content/www/us/en/ark/products/97479/intel-xeon-processor-e3-1270-v6-8m-cache-3-80-ghz.html.
